# Crystal structure of 2,4-di­nitro­phenyl 4-methyl­benzene­sulfonate: a new polymorph

**DOI:** 10.1107/S2056989015015650

**Published:** 2015-08-26

**Authors:** Tyler A. Cooley, Sean Riley, Shannon M. Biros, Richard J. Staples, Felix N. Ngassa

**Affiliations:** a Department of Chemistry, Grand Valley State University, 1 Campus Dr., Allendale, MI 49401, USA; b Center for Crystallographic Research, Department of Chemistry, Michigan State University, 578 S. Shaw Lane, East Lansing, MI, 48824, USA

**Keywords:** crystal structure, aryl sulfonate, π–π inter­actions, C—H⋯O inter­actions, *p*-toluene­sulfonyl chloride

## Abstract

The title compound, C_13_H_10_N_2_O_7_S, was synthesized *via* a nucleophilic substitution reaction between 2,4-di­nitro­phenol and *p*-toluene­sulfonyl chloride. This crystal structure is a polymorph of CSD entry WUVYUH [Vembu *et al.* (2003). *Acta Cryst*, E**59**, o378–380]. The aromatic substituents on the sulfonate group are oriented *gauche* to one another with a C—O—S—C torsion angle of −62.0 (3)°. The supra­molecular features that contribute to the crystal stability are offset π–π [centroid–centroid distance = 3.729 (2) Å] and multiple C—H⋯O inter­actions.

## Abstract 2

The title compound, C_13_H_10_N_2_O_7_S, was solved in the ortho­rhom­bic space group *Pna*2_1_. The aromatic substituents on the sulfonate group are oriented *gauche* to one another with a C—O—S—C torsion angle of −62.0 (3)°. The supra­molecular features that contribute to the crystal lattice are offset π-π and multiple C—H⋯O inter­actions.

## Chemical context   

Nucleophilic substitution reactions at the carbonyl carbon atom are an important class of reactions in biological processes. Analogous to the carbonyl group, nucleophilic substitution reactions of sulfonyl derivatives have also been reported (Castro *et al.*, 2003 ▸; Terrier *et al.*, 2003 ▸; Um *et al.*, 2004 ▸, 2013 ▸; Qrareya *et al.*, 2014 ▸). The mechanism of nucleophilic substitution reactions at the carbonyl group is well understood (Stefanidis *et al.*, 1993 ▸; Lee *et al.*, 2002 ▸). However, the mechanism for nucleophilic substitution reactions at the sulfonyl group is not fully understood (Morales-Rojas & Moss, 2002 ▸; Um *et al.*, 2013 ▸).
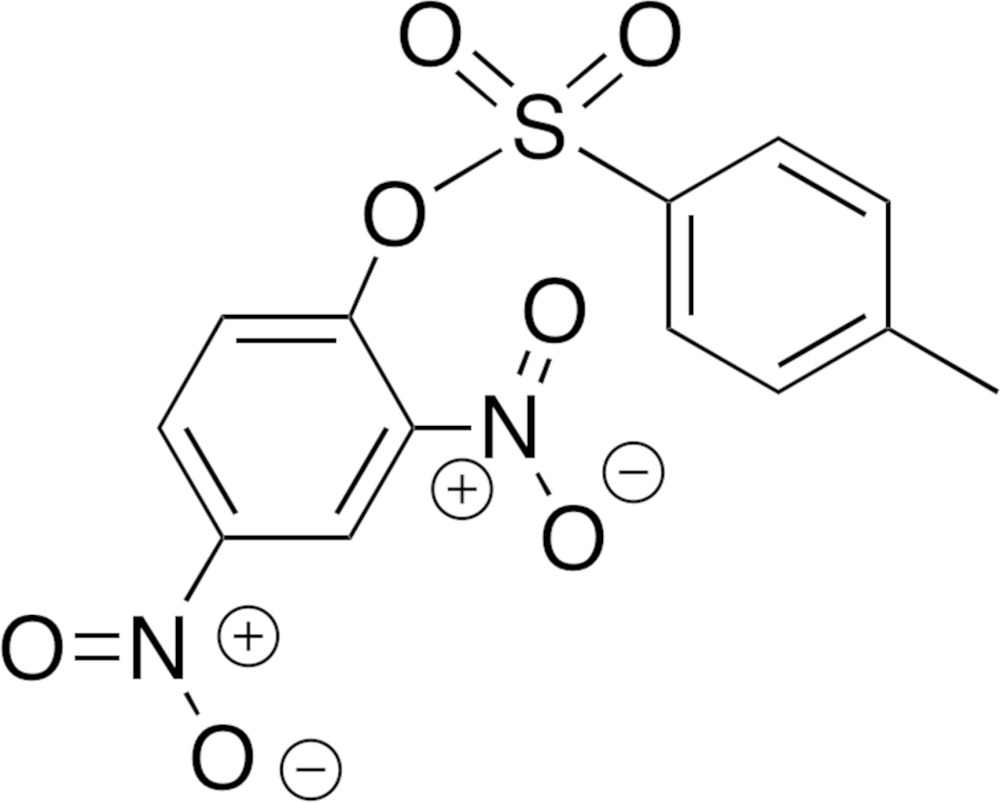


**Figure 1:**
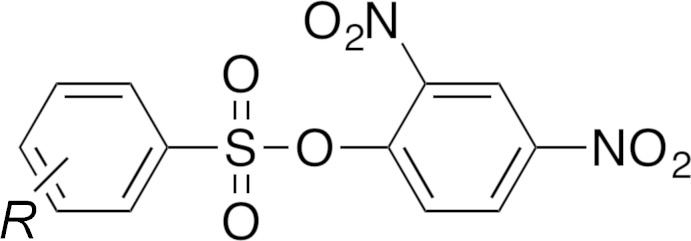
General structure of sulfonate analogues. *R* represents electron-donating and electron-withdrawing substituents.

**Figure 2:**
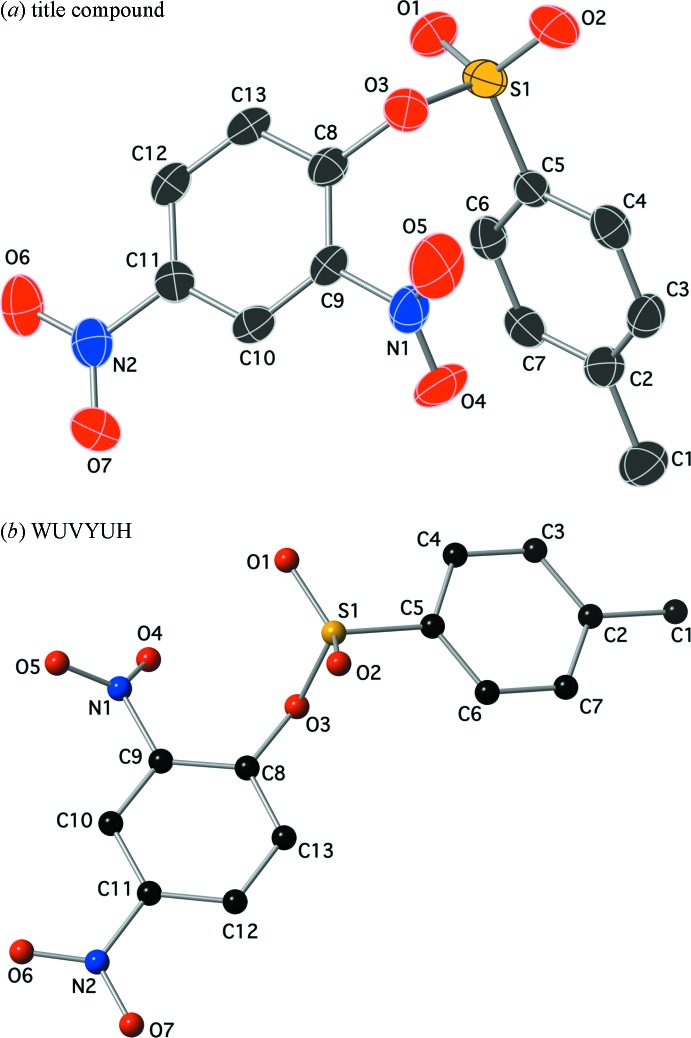
(*a*) The asymmetric unit of the title compound along with the atom-numbering scheme, showing displacement ellipsoids at the 50% probability level; (*b*) the structure and atom-numbering scheme of a polymorph of the title compound WUVYUH (Vembu, *et al.*, 2003*a* ▸). All hydrogen atoms have been omitted for clarity.

A review of the current literature lends credence to both a concerted mechanism and a non-concerted mechanism (Guthrie, 1991 ▸; Colthurst & Williams, 1997 ▸; Spillane *et al.*, 2001 ▸; Um *et al.*, 2003 ▸, 2004 ▸, 2013 ▸). Using primary and secondary amines as nucleophiles, the factors influencing regioselectivity of nucleophilic substitution reactions at the sulfonyl group have been reported (Um *et al.*, 2004 ▸). It has been demonstrated that the regioselectivity and S—O bond-fission mechanism depends on the basicity of the amine and the electronic nature of the sulfonyl substituent. Based on the current state of knowledge in the field, we have sought to capitalize on the chemistry learned on the mechanistic insight of S—O *vs* C—O bond fission by investigating the effect of different substituents on the reactivity of sulfonates. In our work, we are inter­ested in using various sulfonate analogues (Fig. 1 ▸) as electrophilic substrates in nucleophilic aromatic substitution (S_N_Ar) reactions similar to those reported by others (Qrareya *et al.*, 2014 ▸). As the title compound is of inter­est in our ongoing effort on probing the mechanism of S_N_Ar reactions with sulfonate derivatives, we report here on the synthesis and crystal structure of a new polymorph of 2,4-di­nitro­phenyl 4-methyl­benzene­sulfonate (Fig. 2 ▸).

## Structural commentary   

The central sulfur atom (S1) is tetra­hedral with S=O bond lengths of 1.415 (3) and 1.414 (3) Å, and an S—O bond length of 1.634 (3) Å (Fig. 2 ▸ *a*). The bond angle between the S=O groups (O1—S1—O2) is 121.20 (17)°, while that of the aromatic substituents (O3—S1—C5) is 103.27 (15)°. The two aromatic rings are in a *gauche* orientation about the O3—S1 bond with a torsion angle (C8—O3—S1—C5) of −62.0 (3)°.

For comparison, the polymorph WUVYUH (Vembu *et al.*, 2003*a* ▸) has S=O bond lengths of 1.4204 (10) and 1.4246 (10) Å, and the S—O bond length is 1.6195 (9) Å (Fig. 2 ▸ *b*). While the bond lengths of the two polymorphs agree within 0.01 Å of each other, there are some differences between bond angles. The aromatic rings in WUVYUH are in an *anti* orientation along the S—O bond, with a torsion angle of 141.02 (9)°. The bond angle between the S=O groups (O1—S1—O2) is 119.80 (6)°, while that of the aromatic substituents (O3—S1—C5) is 98.17 (5)°.

## Supra­molecular features   

**Figure 3:**
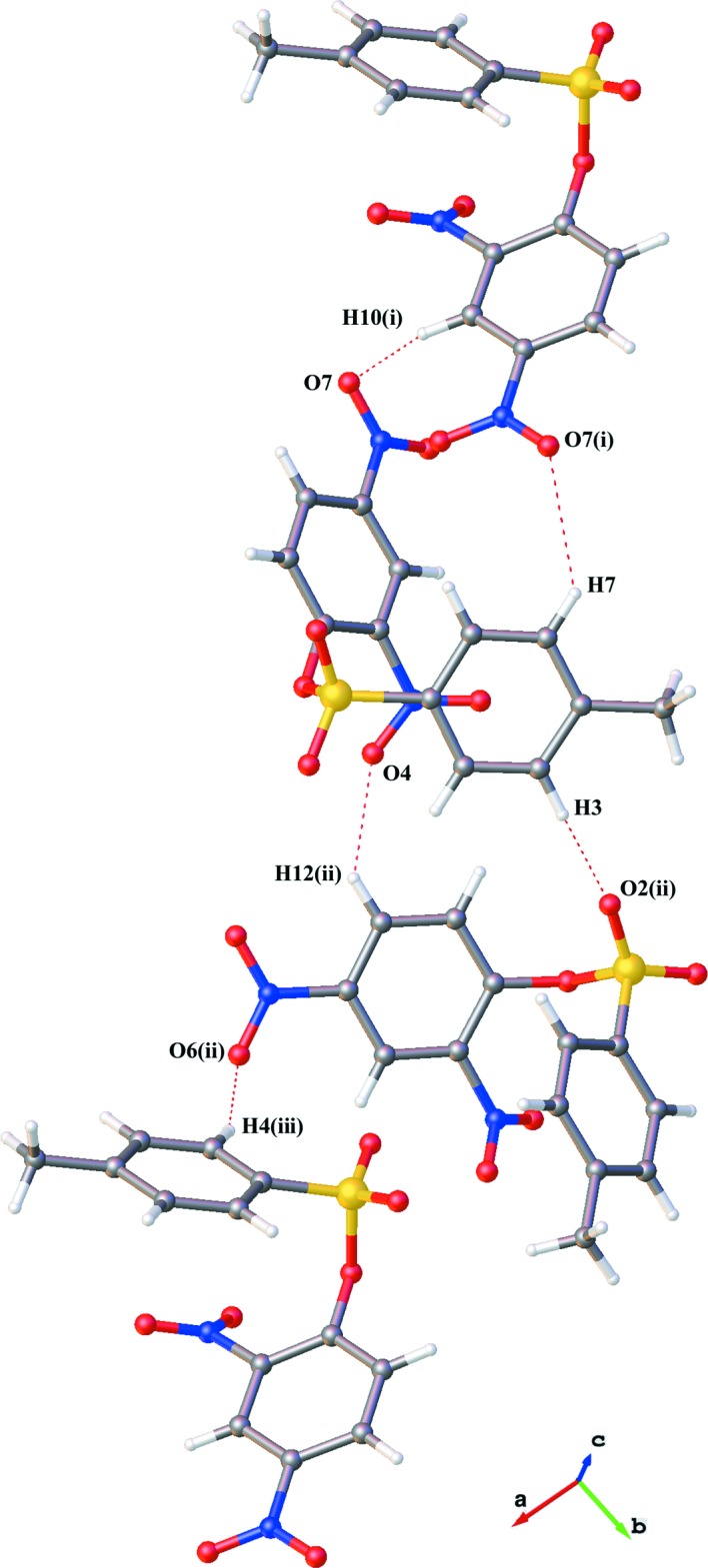
A drawing of a selection of the C—H⋯O inter­actions present in the crystal lattice using a ball and stick model. Symmetry codes: (i) −*x* + 1, −*y* + 1, *z* + 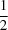; (ii) −*x* + 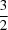, *y* + 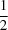, *z* − 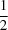; (iii) −*x* + 2, −*y* + 2, *z* − 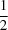.

**Figure 4:**
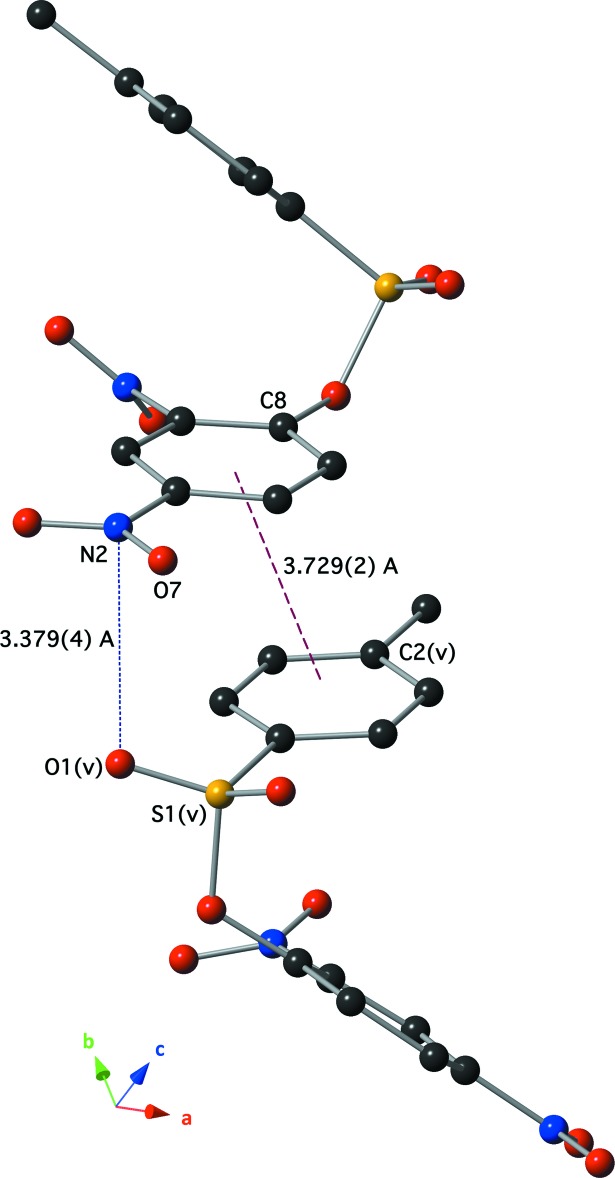
A drawing of the inter­molecular π–π stacking and nitro–sulfonic ester inter­actions present in the crystal using a ball and stick model. Symmetry code (v) −*x* + 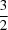, *y* − 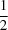, *z* + 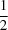.

There are no classical hydrogen-bonding inter­actions in this crystal. There are, however, several inter­molecular C—H⋯O inter­actions with *D*⋯*A* distances less than 3.5 Å and *D*—H⋯*A* angles greater than 120° (Table 1 ▸, Fig. 3 ▸). An offset π–π stacking inter­action is present between the relatively electron-poor ring C8–C13 and the relatively electron-rich ring C2–C7^v^ (Fig. 4 ▸). The centroid–centroid distance is 3.729 (2) Å, the C2–C7 ring is offset by 1.529 (5) Å and tilted 5.74 (12)° out of the plane defined by the C8–C13 ring [symmetry code: (v) 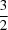 − *x*, −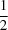 + *y*, −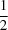 + *z*].

One nitro group (N2,O6,O7) is in proximity to the sulfonic ester of the C2–C7^v^ ring of a nearby π–π dimer. Fig. 4 ▸ also shows that the atoms of these two functional groups are oriented to align the electron-poor N2(nitro) with the electron-rich O1(sulfonic ester), and the electron-poor S1(sulfonic ester) with the electron-rich O7(nitro). Inter­atomic distances are N2(nitro)⋯O17^v^(sulfonic ester) = 3.379 (4) Å, and O7(nitro)⋯S1^v^(sulfonic ester) = 3.877 (3) Å. The relatively short inter­molecular distance between N2 and O17 suggests the presence of favorable N⋯O inter­actions in the crystal (Daszkiewicz, 2013 ▸).

## Database survey   

The Cambridge Structural Database (CSD, version 5.36, May 2015: Groom and Allen, 2014 ▸) contains 171 aromatic 4-toluene­sulfonic esters. Of these, there are 14 structures where the aromatic ring bears substituents at both the 2- and 4-positions. One of these structures is quite similar to the title compound (GOFTIF: Ji *et al.*, 2008 ▸) where the *ortho*-substit­uent is a nitro group and the *para*-position bears a second tosyl­ate group. The remaining entries have various electron-rich groups in the *ortho*-position including meth­oxy (*e.g.* FEMROF: Ichikawa *et al.*, 2004 ▸), eth­oxy (*e.g.* HIRHOG: Ramachandran *et al.*, 2007 ▸), chlorine (OJENEW: Vembu *et al.*, 2003*b* ▸) and alkyl amine (PERFEZ: Zhao *et al.*, 2013 ▸).

The CSD contains three additional structures where the position *ortho* to the sulfonic ester bears a nitro group. In FAYBAJ (Manivannan *et al.*, 2005 ▸), this *o*-nitro group is the only substituent. The aromatic ring in XIYZIP is part of a naphthalene system and also bears a 4-nitro group (Ramachandran *et al.*, 2008 ▸). The third structure in this set is a polymorph of the title compound (WUVYUH: Vembu *et al.*, 2003*a* ▸) that was solved in the ortho­rhom­bic space group *Pbca*. One significant difference between WUVYUH and the title compound is the orientation of the groups around the S—O bond (see the *Structural commentary* section for more details).

## Synthesis and crystallization   

The title compound was prepared by stirring 2,4-di­nitro­phenol (5 mmol), *p*-toluene­sulfonyl chloride (5 mmol) and pyridine (3 mmol) in 10 mL of di­chloro­methane for 30 minutes at room temperature. The reaction was heated to 353 K for 30 minutes in a microwave reactor, then cooled to room temperature and stirred overnight in a fume hood. The reaction mixture was transferred to a scintillation vial where the pale yellow product crystallized upon standing after several days and was filtered from the mother liquor (m.p. 393.4–394.7 K).

## Refinement   

Crystal data, data collection and structure refinement details are summarized in Table 2 ▸. Hydrogen atoms were placed in calculated positions and constrained to ride on their parent atoms, with *U*_iso_(H) = 1.2*U*_eq_(C) for CH groups and *U*_iso_(H) = 1.5 *U*_eq_(C) for methyl groups.

## Supplementary Material

Crystal structure: contains datablock(s) I. DOI: 10.1107/S2056989015015650/pk2562sup1.cif

Structure factors: contains datablock(s) I. DOI: 10.1107/S2056989015015650/pk2562Isup2.hkl

Supporting information file. DOI: 10.1107/S2056989015015650/pk2562Isup3.cml

CCDC reference: 1419864

Additional supporting information:  crystallographic information; 3D view; checkCIF report

## Figures and Tables

**Table 1:** Hydrogen-bond geometry (, )

*D*H*A*	*D*H	H*A*	*D* *A*	*D*H*A*
C3H3O2^i^	0.95	2.42	3.273(5)	149
C4H4O6^ii^	0.95	2.57	3.486(5)	162
C7H7O7^iii^	0.95	2.75	3.499(5)	137
C10H10O7^iv^	0.95	2.51	3.233(5)	133
C12H12O4^v^	0.95	2.34	3.087(5)	135

Symmetry codes: (i) 
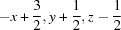
; (ii) 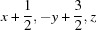; (iii) 
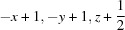
; (iv) 
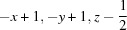
; (v) 
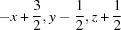
.

**Table 2:** Experimental details

Crystal data	
Chemical formula	C_13_H_10_N_2_O_7_S
*M*_r_	338.29
Crystal system, space group	Orthorhombic, *P* *n* *a*2_1_
Temperature (K)	173
*a*, *b*, *c* ()	14.7716(12), 12.6403(11), 7.6734(6)
*V* (^3^)	1432.8(2)
*Z*	4
Radiation type	Mo *K*
(mm^1^)	0.27
Crystal size (mm)	0.27 0.21 0.20

Data collection	
Diffractometer	Bruker APEXII CCD
Absorption correction	Multi-scan (*SADABS*; Bruker, 2014 ▸)
*T*_min_, *T*_max_	0.686, 0.745
No. of measured, independent and observed [*I* > 2(*I*)] reflections	11432, 2624, 2326
*R*_int_	0.036
(sin /)_max_ (^1^)	0.603

Refinement	
*R*[*F* ^2^ > 2(*F* ^2^)], *wR*(*F* ^2^), *S*	0.034, 0.088, 1.05
No. of reflections	2624
No. of parameters	209
No. of restraints	1
H-atom treatment	H-atom parameters constrained
_max_, _min_ (e ^3^)	0.18, 0.18
Absolute structure	Flack *x* determined using 970 quotients [(*I* ^+^)(*I* )]/[(*I* ^+^)+(*I* )] (Parsons *et al.*, 2013 ▸).
Absolute structure parameter	0.02(4)

Computer programs: *APEX2* and *SAINT* (Bruker, 2013 ▸), *XS* (Sheldrick, 2008 ▸), *SHELXL* (Sheldrick, 2015 ▸), *CrystalMaker* (Palmer, 2007 ▸) and *OLEX2* (Dolomanov *et al.*, 2009 ▸; Bourhis *et al.*, 2015 ▸).

## supplementary crystallographic information

### Crystal data

**Table:**

C_13_H_10_N_2_O_7_S	*D*_x_ = 1.568 Mg m^−^^3^
*M**_r_* = 338.29	Mo *K*α radiation, λ = 0.71073 Å
Orthorhombic, *P**n**a*2_1_	Cell parameters from 5781 reflections
*a* = 14.7716 (12) Å	θ = 2.8–25.4°
*b* = 12.6403 (11) Å	µ = 0.27 mm^−^^1^
*c* = 7.6734 (6) Å	*T* = 173 K
*V* = 1432.8 (2) Å^3^	Chunk, yellow
*Z* = 4	0.27 × 0.21 × 0.20 mm
*F*(000) = 696	

### Data collection

**Table:**

Bruker APEXII CCD diffractometer	2624 independent reflections
Radiation source: sealed tube	2326 reflections with *I* > 2σ(*I*)
Graphite monochromator	*R*_int_ = 0.036
Detector resolution: 8 pixels mm^-1^	θ_max_ = 25.4°, θ_min_ = 2.1°
φ and ω scans	*h* = −17→17
Absorption correction: multi-scan (*SADABS*; Bruker, 2014)	*k* = −15→15
*T*_min_ = 0.686, *T*_max_ = 0.745	*l* = −9→9
11432 measured reflections	

### Refinement

**Table:**

Refinement on *F*^2^	Hydrogen site location: inferred from neighbouring sites
Least-squares matrix: full	H-atom parameters constrained
*R*[*F*^2^ > 2σ(*F*^2^)] = 0.034	*w* = 1/[σ^2^(*F*_o_^2^) + (0.0448*P*)^2^ + 0.2683*P*] where *P* = (*F*_o_^2^ + 2*F*_c_^2^)/3
*wR*(*F*^2^) = 0.088	(Δ/σ)_max_ < 0.001
*S* = 1.05	Δρ_max_ = 0.18 e Å^−^^3^
2624 reflections	Δρ_min_ = −0.18 e Å^−^^3^
209 parameters	Absolute structure: Flack *x* determined using 970 quotients [(*I*^+^)-(*I*^-^)]/[(*I*^+^)+(*I*^-^)] (Parsons *et al.*, 2013).
1 restraint	Absolute structure parameter: −0.02 (4)

### Special details

**Table:**

Experimental. SADABS (Bruker, 2014) was used for absorption correction. wR2(int) was 0.0928 before and 0.0535 after correction. The Ratio of minimum to maximum transmission is 0.9202. The λ/2 correction factor is 0.00150.
Geometry. All e.s.d.'s (except the e.s.d. in the dihedral angle between two l.s. planes) are estimated using the full covariance matrix. The cell e.s.d.'s are taken into account individually in the estimation of e.s.d.'s in distances, angles and torsion angles; correlations between e.s.d.'s in cell parameters are only used when they are defined by crystal symmetry. An approximate (isotropic) treatment of cell e.s.d.'s is used for estimating e.s.d.'s involving l.s. planes.

### Fractional atomic coordinates and isotropic or equivalent isotropic displacement parameters (Å^2^)

**Table:**

	*x*	*y*	*z*	*U*_iso_*/*U*_eq_	
S1	0.84591 (5)	0.72917 (7)	0.62435 (14)	0.0377 (2)	
O1	0.92559 (18)	0.7834 (2)	0.5749 (4)	0.0520 (8)	
O2	0.84576 (18)	0.6573 (2)	0.7657 (4)	0.0480 (8)	
O3	0.82059 (18)	0.6622 (2)	0.4489 (3)	0.0393 (6)	
O4	0.7331 (2)	0.7555 (3)	0.1741 (4)	0.0698 (10)	
O5	0.6041 (2)	0.8003 (2)	0.2866 (5)	0.0622 (9)	
O6	0.43774 (19)	0.4573 (2)	0.3474 (4)	0.0569 (8)	
O7	0.5026 (2)	0.3344 (2)	0.4987 (4)	0.0552 (8)	
N1	0.6675 (2)	0.7407 (3)	0.2684 (5)	0.0447 (8)	
N2	0.5033 (2)	0.4212 (3)	0.4265 (4)	0.0409 (7)	
C1	0.5319 (3)	1.0307 (3)	0.6779 (6)	0.0526 (11)	
H1A	0.4769	0.9950	0.6376	0.079*	
H1B	0.5436	1.0927	0.6047	0.079*	
H1C	0.5240	1.0531	0.7991	0.079*	
C2	0.6109 (2)	0.9555 (3)	0.6659 (5)	0.0383 (9)	
C3	0.6881 (3)	0.9821 (3)	0.5730 (5)	0.0391 (9)	
H3	0.6911	1.0490	0.5167	0.047*	
C4	0.7608 (2)	0.9138 (3)	0.5602 (4)	0.0360 (8)	
H4	0.8130	0.9328	0.4950	0.043*	
C5	0.7559 (2)	0.8168 (2)	0.6448 (4)	0.0299 (7)	
C6	0.6796 (2)	0.7879 (3)	0.7391 (4)	0.0345 (8)	
H6	0.6770	0.7214	0.7966	0.041*	
C7	0.6077 (2)	0.8575 (3)	0.7480 (5)	0.0373 (8)	
H7	0.5551	0.8380	0.8114	0.045*	
C8	0.7418 (2)	0.6020 (3)	0.4478 (5)	0.0323 (8)	
C9	0.6651 (2)	0.6401 (3)	0.3637 (4)	0.0307 (7)	
C10	0.5859 (2)	0.5833 (3)	0.3596 (5)	0.0337 (8)	
H10	0.5329	0.6110	0.3061	0.040*	
C11	0.5861 (2)	0.4846 (3)	0.4361 (5)	0.0323 (8)	
C12	0.6613 (2)	0.4440 (3)	0.5188 (5)	0.0363 (8)	
H12	0.6593	0.3754	0.5693	0.044*	
C13	0.7395 (2)	0.5035 (3)	0.5279 (5)	0.0362 (8)	
H13	0.7911	0.4774	0.5880	0.043*	

### Atomic displacement parameters (Å^2^)

**Table:**

	*U*^11^	*U*^22^	*U*^33^	*U*^12^	*U*^13^	*U*^23^
S1	0.0284 (4)	0.0403 (4)	0.0443 (5)	0.0001 (4)	−0.0037 (5)	0.0048 (5)
O1	0.0275 (12)	0.0566 (16)	0.072 (2)	−0.0066 (12)	0.0026 (13)	0.0053 (15)
O2	0.0478 (17)	0.0453 (15)	0.0507 (17)	0.0058 (12)	−0.0094 (13)	0.0130 (13)
O3	0.0330 (13)	0.0407 (14)	0.0444 (15)	0.0004 (12)	0.0077 (11)	−0.0014 (12)
O4	0.077 (2)	0.069 (2)	0.063 (2)	−0.0155 (17)	0.0088 (18)	0.0306 (17)
O5	0.073 (2)	0.0364 (14)	0.077 (2)	0.0159 (15)	−0.0244 (17)	0.0065 (15)
O6	0.0387 (16)	0.0549 (17)	0.077 (2)	−0.0012 (14)	−0.0106 (16)	−0.0068 (17)
O7	0.070 (2)	0.0441 (15)	0.0517 (17)	−0.0187 (14)	0.0021 (15)	0.0053 (14)
N1	0.058 (2)	0.0326 (16)	0.044 (2)	−0.0040 (16)	−0.0153 (17)	0.0052 (14)
N2	0.044 (2)	0.0391 (16)	0.0391 (18)	−0.0051 (15)	0.0050 (16)	−0.0065 (15)
C1	0.051 (2)	0.047 (2)	0.059 (3)	0.0125 (18)	−0.009 (2)	−0.011 (2)
C2	0.0358 (19)	0.0371 (18)	0.042 (2)	0.0007 (16)	−0.0077 (16)	−0.0076 (17)
C3	0.050 (2)	0.0285 (17)	0.039 (2)	−0.0011 (16)	−0.0090 (17)	0.0033 (15)
C4	0.0358 (19)	0.0388 (19)	0.0335 (19)	−0.0086 (16)	0.0015 (15)	0.0028 (15)
C5	0.0268 (16)	0.0322 (15)	0.0307 (18)	−0.0046 (13)	−0.0045 (15)	0.0029 (17)
C6	0.037 (2)	0.0323 (18)	0.0342 (19)	−0.0063 (15)	−0.0001 (16)	0.0020 (16)
C7	0.0304 (18)	0.042 (2)	0.039 (2)	−0.0069 (16)	0.0000 (16)	−0.0065 (17)
C8	0.0294 (18)	0.0328 (18)	0.0346 (17)	0.0035 (14)	0.0080 (15)	0.0015 (16)
C9	0.0383 (19)	0.0254 (16)	0.0283 (16)	0.0049 (15)	0.0017 (15)	0.0020 (14)
C10	0.036 (2)	0.0325 (17)	0.0324 (18)	0.0090 (15)	−0.0008 (16)	−0.0015 (15)
C11	0.0328 (19)	0.0326 (17)	0.0315 (18)	−0.0003 (15)	0.0062 (15)	−0.0024 (15)
C12	0.044 (2)	0.0280 (17)	0.0366 (19)	0.0059 (15)	0.0014 (17)	0.0056 (15)
C13	0.0362 (19)	0.0326 (17)	0.0397 (19)	0.0112 (15)	0.0006 (16)	0.0040 (16)

### Geometric parameters (Å, º)

**Table:**

S1—O1	1.414 (3)	C3—H3	0.9500
S1—O2	1.415 (3)	C3—C4	1.381 (5)
S1—O3	1.634 (3)	C4—H4	0.9500
S1—C5	1.737 (3)	C4—C5	1.390 (4)
O3—C8	1.391 (4)	C5—C6	1.388 (5)
O4—N1	1.224 (4)	C6—H6	0.9500
O5—N1	1.210 (4)	C6—C7	1.381 (5)
O6—N2	1.231 (4)	C7—H7	0.9500
O7—N2	1.228 (4)	C8—C9	1.389 (5)
N1—C9	1.468 (5)	C8—C13	1.389 (5)
N2—C11	1.465 (4)	C9—C10	1.374 (5)
C1—H1A	0.9800	C10—H10	0.9500
C1—H1B	0.9800	C10—C11	1.378 (5)
C1—H1C	0.9800	C11—C12	1.379 (5)
C1—C2	1.507 (5)	C12—H12	0.9500
C2—C3	1.387 (5)	C12—C13	1.379 (5)
C2—C7	1.391 (5)	C13—H13	0.9500
			
O1—S1—O2	121.20 (17)	C4—C5—S1	118.8 (3)
O1—S1—O3	102.73 (16)	C6—C5—S1	120.0 (2)
O1—S1—C5	110.64 (16)	C6—C5—C4	121.2 (3)
O2—S1—O3	107.40 (14)	C5—C6—H6	120.6
O2—S1—C5	109.80 (17)	C7—C6—C5	118.8 (3)
O3—S1—C5	103.27 (15)	C7—C6—H6	120.6
C8—O3—S1	118.7 (2)	C2—C7—H7	119.4
O4—N1—C9	116.5 (3)	C6—C7—C2	121.3 (3)
O5—N1—O4	125.9 (3)	C6—C7—H7	119.4
O5—N1—C9	117.6 (4)	C9—C8—O3	119.7 (3)
O6—N2—C11	118.6 (3)	C13—C8—O3	120.6 (3)
O7—N2—O6	123.2 (3)	C13—C8—C9	119.8 (3)
O7—N2—C11	118.3 (3)	C8—C9—N1	120.8 (3)
H1A—C1—H1B	109.5	C10—C9—N1	117.5 (3)
H1A—C1—H1C	109.5	C10—C9—C8	121.6 (3)
H1B—C1—H1C	109.5	C9—C10—H10	121.3
C2—C1—H1A	109.5	C9—C10—C11	117.5 (3)
C2—C1—H1B	109.5	C11—C10—H10	121.3
C2—C1—H1C	109.5	C10—C11—N2	118.2 (3)
C3—C2—C1	121.0 (3)	C10—C11—C12	122.3 (3)
C3—C2—C7	118.5 (3)	C12—C11—N2	119.5 (3)
C7—C2—C1	120.5 (3)	C11—C12—H12	120.2
C2—C3—H3	119.2	C11—C12—C13	119.7 (3)
C4—C3—C2	121.6 (3)	C13—C12—H12	120.2
C4—C3—H3	119.2	C8—C13—H13	120.5
C3—C4—H4	120.7	C12—C13—C8	119.1 (3)
C3—C4—C5	118.6 (3)	C12—C13—H13	120.5
C5—C4—H4	120.7		
			
S1—O3—C8—C9	101.6 (3)	O7—N2—C11—C12	2.6 (5)
S1—O3—C8—C13	−79.5 (4)	N1—C9—C10—C11	174.5 (3)
S1—C5—C6—C7	−177.7 (3)	N2—C11—C12—C13	179.9 (3)
O1—S1—O3—C8	−177.1 (2)	C1—C2—C3—C4	−179.7 (3)
O1—S1—C5—C4	21.1 (3)	C1—C2—C7—C6	−179.7 (4)
O1—S1—C5—C6	−161.2 (3)	C2—C3—C4—C5	−0.7 (5)
O2—S1—O3—C8	54.0 (3)	C3—C2—C7—C6	0.5 (5)
O2—S1—C5—C4	157.5 (3)	C3—C4—C5—S1	178.3 (3)
O2—S1—C5—C6	−24.8 (3)	C3—C4—C5—C6	0.6 (5)
O3—S1—C5—C4	−88.2 (3)	C4—C5—C6—C7	0.0 (5)
O3—S1—C5—C6	89.5 (3)	C5—S1—O3—C8	−62.0 (3)
O3—C8—C9—N1	3.0 (5)	C5—C6—C7—C2	−0.6 (5)
O3—C8—C9—C10	−179.9 (3)	C7—C2—C3—C4	0.2 (5)
O3—C8—C13—C12	−177.6 (3)	C8—C9—C10—C11	−2.7 (5)
O4—N1—C9—C8	44.8 (5)	C9—C8—C13—C12	1.4 (5)
O4—N1—C9—C10	−132.4 (4)	C9—C10—C11—N2	−177.5 (3)
O5—N1—C9—C8	−136.9 (4)	C9—C10—C11—C12	1.9 (5)
O5—N1—C9—C10	46.0 (5)	C10—C11—C12—C13	0.5 (5)
O6—N2—C11—C10	1.6 (5)	C11—C12—C13—C8	−2.2 (5)
O6—N2—C11—C12	−177.8 (3)	C13—C8—C9—N1	−176.0 (3)
O7—N2—C11—C10	−178.0 (3)	C13—C8—C9—C10	1.1 (5)

### Hydrogen-bond geometry (Å, º)

**Table:**

*D*—H···*A*	*D*—H	H···*A*	*D*···*A*	*D*—H···*A*
C3—H3···O2^i^	0.95	2.42	3.273 (5)	149
C4—H4···O6^ii^	0.95	2.57	3.486 (5)	162
C7—H7···O7^iii^	0.95	2.75	3.499 (5)	137
C10—H10···O7^iv^	0.95	2.51	3.233 (5)	133
C12—H12···O4^v^	0.95	2.34	3.087 (5)	135

Symmetry codes: (i) −*x*+3/2, *y*+1/2, *z*−1/2; (ii) *x*+1/2, −*y*+3/2, *z*; (iii) −*x*+1, −*y*+1, *z*+1/2; (iv) −*x*+1, −*y*+1, *z*−1/2; (v) −*x*+3/2, *y*−1/2, *z*+1/2.
